# NetFACS: Using network science to understand facial communication systems

**DOI:** 10.3758/s13428-021-01692-5

**Published:** 2021-11-09

**Authors:** Alexander Mielke, Bridget M. Waller, Claire Pérez, Alan V. Rincon, Julie Duboscq, Jérôme Micheletta

**Affiliations:** 1 Department of Psychology, Centre for Comparative and Evolutionary Psychology, University of Portsmouth, King Henry I Street, Portsmouth, PO1 2DY UK; 2 Primate Models for Behavioural Evolution Lab, School of Anthropology and Museum Ethnography, University of Oxford, 64 Banbury Road, Oxford, OX2 6PN UK; 3 Department of Psychology, Nottingham Trent University, Nottingham, UK; 4 UMR7206 Eco-Anthropology, CNRS-MNHN-Université de Paris, Paris, France

**Keywords:** Facial action coding system, Facial signals, Network analysis, Communication

## Abstract

Understanding facial signals in humans and other species is crucial for understanding the evolution, complexity, and function of the face as a communication tool. The Facial Action Coding System (FACS) enables researchers to measure facial movements accurately, but we currently lack tools to reliably analyse data and efficiently communicate results. Network analysis can provide a way to use the information encoded in FACS datasets: by treating individual AUs (the smallest units of facial movements) as nodes in a network and their co-occurrence as connections, we can analyse and visualise differences in the use of combinations of AUs in different conditions. Here, we present ‘NetFACS’, a statistical package that uses occurrence probabilities and resampling methods to answer questions about the use of AUs, AU combinations, and the facial communication system as a whole in humans and non-human animals. Using highly stereotyped facial signals as an example, we illustrate some of the current functionalities of NetFACS. We show that very few AUs are specific to certain stereotypical contexts; that AUs are not used independently from each other; that graph-level properties of stereotypical signals differ; and that clusters of AUs allow us to reconstruct facial signals, even when blind to the underlying conditions. The flexibility and widespread use of network analysis allows us to move away from studying facial signals as stereotyped expressions, and towards a dynamic and differentiated approach to facial communication.

## Introduction

Facial signals are important social communication tools in many species, including humans (Crivelli & Fridlund, 2018; Jack & Schyns, 2015; Waller & Micheletta, 2013). On their own and when accompanying other signals, such as speech and gestures, they can convey information to others about the sender’s motivation and future behaviour (Parkinson, 2005; Waller et al., 2017). Studies of human facial signals, however, have traditionally focused strongly on their connections to a small number of emotions (Darwin, 1872; Ekman, 2003). This focus is linked to a common research paradigm: a set of stereotypical facial signals, often posed by trained models, is presented to participants, together with a finite number of possible meanings (Ekman & Friesen, 1986). Alternatively, emotions are induced experimentally and the resulting facial signals analysed (Keltner, 1995). Situational variables are largely removed, and stereotypical facial signals are assumed to have one distinct function across contexts (Jack & Schyns, 2015). Studies of facial signals in natural contexts are rare (Crivelli & Fridlund, 2018). Facial signals are therefore not currently studied like other communication systems: To understand how humans or other animals use vocal communication, for example, researchers often collect signals produced naturally, identify meaningful units, and test how reliably units are used and linked to different contexts (Fischer & Price, 2017; Kershenbaum et al., 2016). ‘Meaning’ in this context is defined by the specificity of signal production and response by receivers (Kershenbaum et al., 2016). Production is ‘specific’ if the signal is associated with one context and reliably differentiates it from other contexts. To understand the use of facial behaviour as a communication system beyond a small set of discrete signals, we need an interaction-centred approach that can detect the underlying structure of facial communication, using a wide range of movements. Otherwise, the complexity of facial communication systems is overlooked.

The face is capable of producing complex, coordinated movements, where the relationship between muscles is important, and it is possible that complexity is reflected in the quantity and quality of these relationships. Facial signals are produced largely due to the coordinated action of facial muscle movements. The human face has approximately 20 independent muscles (see Burrows, 2008) which are capable of causing subtle changes to facial landmarks. The muscles have discrete attachment sites to bone or soft tissue and most can operate independently, occasionally with multiple different movements producible from the same muscle. The volume of the primate facial nuclei of the brainstem is large in comparison to other mammals, suggesting that primates have evolved greater motor control of facial muscles (Sherwood, 2005) and greater capacity to operate them independently of each other. Human facial muscles have a high degree of slow twitch muscle fibre (Burrows et al., 2014), meaning that these muscles are capable of controlled, sustained contraction. Some authors have conceptualised ‘modules’ in head/neck anatomy, with the physical connections between structures (including specific facial muscles) being stronger or weaker depending on how they function together (Esteve-Altava et al., 2015).

Facial signals are often coded using the Facial Action Coding System (FACS; Ekman et al., 2002; Ekman & Friesen, 1978). In the FACS, ‘action units’ (AU) are considered the smallest unit of facial communication. They are based largely on visible facial muscle activity, and as a system are meant to cover all possible movements individuals can show using their face. AUs are classified as active or not active (i.e., muscle movement is visible or not), either for a signal in its entirety (e.g., if photos are coded or videos summarised), or for a specific period in a video (Ekman & Friesen, 1978). Intensity evaluation of AU usage (e.g., on a five-point scale) is possible. This system allows for reliable classifications of facial signals: for example, instead of saying that a participant ‘smiled’, we can characterise the facial signal by AUs 6 and 12, and the duration and intensity of each of these can be determined. Importantly, this system enables the analysis of facial signals that cannot be as easily categorised as ‘smile’ or ‘frown’. It also emphasises the potential complexity of facial communication: AUs can occur on their own or with others—the 27 main AU codes of the FACS can theoretically be combined in more than 8 billion ways. These combinations are connected dynamically in sequences, further increasing the potential for transmitting detailed information. Over the years, FACS has been developed for a number of non-human species (orangutans: Caeiro et al., 2013; cats: Caeiro et al., 2017; Barbary macaques: Julle-Danière et al., 2015; rhesus macaques: Parr et al., 2010; chimpanzees: Vick et al., 2007; gibbons: Waller et al., 2012; dogs: Waller et al., 2013a, b; horses: Wathan et al., 2015). This allows for detailed and objective comparative research (Julle-Danière et al., 2015; Scheider et al., 2014, 2016; Waller et al., 2020).

Historically, many studies have taken an all-or-nothing approach to facial signals by claiming that certain combinations of AU form a ‘fear’ face or an ‘angry’ face, so the AUs constituting these signals are deterministically linked to that emotion (Ekman & Oster, 1979; Matsumoto et al., 2008). However, given that some AUs are shared even between basic ‘emotion’ signals, some AUs are not used in any of them, and signals have meaningful dynamic features (Jack et al., 2014; Krumhuber et al., 2013), it is likely that the signal value of AUs and AU combinations is probabilistic (Crivelli & Fridlund, 2018). In other communication systems, the same signal element can often have different functions based on the context (e.g., Abramson et al., 2020; Aviezer et al., 2008); at the same time, different signals can have the same function (Isac & Reiss, 2008). The strong focus on few stereotypical facial signals has prevented us from understanding whether the same is true for facial signals. With the development of automated FACS-coding(e.g., Lewinski et al., 2014), large datasets of diverse facial signals will become increasingly important, but the development of coding software has outpaced the development of appropriate statistical tools to interpret resulting data.

Most studies try to establish how much the use of each AU differs across conditions, such as different social contexts or internal experience of participants. However, currently, many different approaches are in circulation, reducing reproducibility, and FACS datasets have some features that make traditional statistical models unsuitable for this task. FACS data mix spatial and temporal combinations of AUs, with temporal information influencing comprehension (Krumhuber et al., 2013); different signals are also strung together in sequences, often combined with speech or gestures (Kessous et al., 2010). At a basic level, for each data point in FACS coding (either a static description, or a time point in a dynamic analysis), AUs are either present or absent (or categorical if intensity measures are provided). When testing whether the mean use of an AU across data points differs between conditions, many analyses of FACS data are based on analysis of variance (e.g., Harris & Alvarado, 2005). When testing which AUs occur together in specific contexts, researchers often use dimension reduction techniques such as principal component analysis or factor analysis (e.g., Stratou et al., 2017). As FACS data are inherently binomial (AUs are either there or not), categorical (if coded using intensity measures), or proportional (proportion of events in which AU is active), they violate distributional assumptions of these methods. Additionally, the use of different AUs does not have to follow the same distribution—for example, if only a subset of individuals in a population use some AUs while others do not, or an AU is occasionally combined with another to change meaning, the distribution of that AU in the population would be bimodal. FACS datasets contain dependencies between units and events on different levels: for example, participants often provide multiple videos. Muscle movements throughout a video are not independent from each other. Participants often share many characteristics (e.g., gender, place of origin, age, etc.) that can influence facial signals (Jack et al., 2012). All of these levels of dependence need to be accounted for so as to avoid pseudoreplication (Hurlbert, 1984; Waller, Warmelink, et al., 2013b). Logistic regression would be capable of modelling the impact of these characteristics on single AU movements and control for temporal autocorrelation. However, AUs themselves do not act independently from each other: almost no facial signal consists of a single muscle moving (Krumhuber & Scherer, 2011). This raises the possibility that AUs are not the smallest unit of facial communication, and that the combination of AUs rather than their individual occurrence is of relevance. Structural equation modelling, if extended for categorical variables (Kupek, 2006), could potentially satisfy some of these requirements, but would require large amounts of data given the number of AUs of interest.

The role of AU usage in facial signal perception has been described in detail, including using probabilistic and information theoretical approaches (Jack & Schyns, 2017), notably nonnegative matrix factorisation to decompose dynamic interactions between different AUs (Delis et al., 2016). To move towards an interactional approach, removing the need for recipient judgement, we propose the use of a method that has been successfully applied to vocal communication, both in humans and non-human animals, in an attempt to enable researchers to study facial communication as a communication system like others. Network theory has been used to understand the co-occurrence of syllables and words in different languages as a measure of how similar languages are (Baronchelli et al., 2013; Čech et al., 2011; Ferrer i Cancho et al., 2004; Liu & Xu, 2011). For example, the ‘Netlang’ software package (Barceló-Coblijn et al., 2017) creates networks of words in a corpus while controlling for their syntactic relations. Networks have also been applied to temporal information in animal vocalisations (Kershenbaum et al., 2016), elucidating transitions between call types in songbirds (Deslandes et al., 2014; Hedley, 2016; Sasahara et al., 2012; Weiss et al., 2014) and humpback whales (Allen et al., 2019). The musculature underlying facial movements has been described in terms of networks of muscles (Esteve-Altava et al., 2015). Dynamic Bayesian networks of AU co-occurrence probabilities have been used to improve correct automated identification of AU intensity from video data (Li et al., 2015; Tong et al., 2007). Analysing FACS data as networks allows for direct comparisons with other communication systems with regard to their complexity and information content (Lynn et al., 2020). Network analysis can answer questions on different analytical levels (Newman, 2003): AUs themselves, their combinations, full facial signals, and the face as a whole (Baronchelli et al., 2013). Networks of transitions between AUs or AU combinations can illuminate the dynamics of facial signals. Therefore, applying network science to facial communication using AUs promises to increase our ability to understand facial signals beyond a small number of highly stereotyped signals, and accordingly, ask new questions about facial signals as an evolved communication system.

Network approaches are useful because they are highly flexible: potentially communicative units (such as syllables or AUs) are used as ‘nodes’, which are linked by ‘edges’ reflecting different types of directed and undirected connections (Baronchelli et al., 2013; Newman, 2010). For example, the edge between two AUs can represent the probability that they will occur together in a static image, or that one will occur after the other in a discrete sequence (Kershenbaum et al., 2016). Because networks are used in a vast number of fields (Newman, 2010), standardised methods exist to address a wide variety of questions in a statistically appropriate manner. Results can be displayed using network graphs, which are more intuitive to readers than the tables of AU combinations that often accompany research articles using FACS. This solves one of the central problems of FACS research so far: the large number of elements makes it unwieldy to describe the behaviour of all possible combinations in writing (Scherer et al., 2019). Centrality measures can quantify the position of each node in the network, and characterise the network as a whole (Milo et al., 2002). Fundamentally, these approaches rely on the creation of appropriate null models to address the question at hand, enabling researchers to account for the underlying data structure, autocorrelation, and potentially important control variables (Farine & Whitehead, 2015).

In this article, we set out to present the potential power of using network analysis to understand and visualise detailed facial signal data. To demonstrate the fundamentals of the method to a wide audience, we use a simple dataset of stereotypical static signals, while the true potential for network analysis in this context lies in applying it to ‘natural’ datasets of non-posed facial signals with temporal information. For many researchers working with facial signal data, networks are not part of the toolset they apply regularly. We have therefore developed the R package ‘NetFACS’ (https://github.com/NetFACS/NetFACS) that facilitates the application of the methods described in this article. We will use network measures to address questions on different levels: (a) How specific are AUs to certain stereotypical signals (unit level)? (b) Which AUs are conditional on each other (dyad level)? (c) Can we reconstruct the stereotypical facial signals for each context through dyadic networks, and how do networks in different contexts compare (signal level)? (d) Can we identify clusters of AUs without knowledge of the underlying contexts (system level)? Ultimately, the goal of NetFACS is to create a standardised way to analyse facial signals to determine how the face as a communication system compares to other systems in humans and other species. We developed it to quantify the information contained in the facial signals of different species, going beyond the simplistic use of a small set of highly stereotyped signals. This will allow us to address questions regarding the evolution of the use of facial signals in different species in different conditions, inter-individual differences and flexibility in signal use within a species, but also the complexity of facial communication systems across species.

## Methods

### Glossary

**Table:** 

*Term*	*Definition*
Action unit	Smallest unit of visible facial movement, based on underlying muscle distribution
Unconditional probability	Proportion of events in which a unit or combination of units occurs in a condition or dataset: $P(A)=\frac{Number\ of\ occurrences\ of\ A}{Number\ of\ possible\ occurrences\ of\ A}$
Conditional probability	Proportion of events in which a unit occurs, given that another unit is already present: $P\left(B|A\right)=\frac{Number\ of\ occurrences\ of\ A\ and\ B\ together}{Number\ of\ occurrences\ of\ A}$
Specificity	Proportion of events in which a condition is observed if an element is present; strength of evidence that AU is connected to a condition rather than another: $P\left(C1|A\right)=\frac{Number\ of\ occurrences\ of\ A\ in\ Context\ 1}{Number\ of\ total\ occurrences\ of\ A}$
Bootstrap	A resampling method in which cases of a condition are repeatedly randomly selected with replacement and the statistic of interest is calculated for each iteration, to provide estimates of the distribution of the statistic in the sample
Permutation	A resampling method in which the null distribution of a statistic of interest is created by randomly shuffling some aspect of the original distribution repeatedly
Node	Element of the network, in this case the AUs
Edge	Connection between elements/nodes in a network; e.g., based on the co-occurrence of AUs or on their conditional probability
Weighted network	Network in which the edges between nodes can take different values depending on how weakly or strongly the nodes are connected
Unweighted network	Network in which the edges between nodes are either 1 (present) or 0 (absent)
Directed network	Network in which the connections between two nodes can be asymmetrical, i.e., *A* → *B* ≠ *B* → *A*
Undirected network	Network in which the connections between two nodes is symmetrical, i.e., *A* ↔ *B*
Bipartite graph	Network in which nodes from two different categories (e.g., condition and AU) can be connected between categories (*AU*→ Condition), but not within categories
Node cluster	Structural element of a network, group of nodes that have strong connection with each other, but weak connections with other nodes outside the cluster
Transitivity	Triads of nodes are considered transitive if all three nodes are connected with each other; a network has high transitivity if a large number of triads are closed ( *A* → *B* → *C* → *A*) compared to triads that just show two connections (e.g. *A* → *B*, *B* → *C*)
Density	Proportion of the potential connections between nodes that are actual existing connections. In a dense network, all or most connections are present; in a sparse network, only few connections are present
Degree	Number of actual connections a node has with other nodes in a network. Nodes with high degree co-occur frequently with other nodes
Strength	Mean weight of connections of a node with all other nodes in a weighted network

### Dataset

For this study, we used the peak frames of 327 FACS-coded videos assigned to one of seven conditions (anger: 45 images, contempt: 18, disgust: 59, fear: 25, happy: 69, sadness: 28, surprise: 83) in the Extended Cohn-Kanade Database (Kanade et al., 2000; Lucey et al., 2010). Videos included only one facial signal, produced based on instructions from an experimenter, and were specifically designed as a baseline dataset for automated facial expression detection (Kanade et al., 2000). Images represented a subset of posed facial expressions judged to fit the stereotypical expressions for those conditions (e.g., AU6+12 for a ‘happy’ face). Thus, the dataset is designed to reduce variability within conditions and increase variability between conditions, making the specificity of AUs unnaturally high; however, this suits our purpose of presenting the method in a clear-cut example. In the following, we will talk about the emotion labels that were assigned to each video as experimental ‘conditions’, to make clear that while here we use the labels as an example, researchers could also compare other conditions with each other, e.g., participant gender. There is no sequential information available in this database, and we omit information about intensity of AU activity as NetFACS is currently designed to work on presence/absence information only. Data including intensity measures can easily be adapted to represent presence/absence. Data were organised so that each image (subsequently ‘event’) is one row, and each AU is a column that is either present (1) or absent (0). As no ‘neutral’ condition is available, all analyses compare the data for each of the conditions against all the other conditions. Of the original 23 AUs in the Extended Cohn-Kanade database, AU11, 21, and 22 did not occur more than once in this subset so they were removed. We also dropped AU25 because it can be the result of different muscle movements.

### Probability

All networks in NetFACS are built based on the probability of AU occurrence and co-occurrence of multiple AUs. This builds on previous work using a probabilistic representation of AU co-occurrence(Li et al., 2015). Co-occurrence of communicative units is an important feature in language learning: repeated coupling of units enables infants to learn word composition and syntactic rules (Isac & Reiss, 2008). When analysing units of facial communication (either AUs, combinations of AUs, or whole signals), we are interested in the *unconditional probability* that they occur and co-occur with each other. The unconditional probability for FACS-like data represents the number of events in which an AU or combination was active, given the total number of events: so, if an AU is active in 5% of all events, its unconditional probability is 0.05. At the same time, we are interested in the *conditional probability*: the proportion of events in which units occur when another one is also present, or occur if a specific condition is given. The difference between unconditional and conditional probability is important to understand how AUs tie to each other: for example, the combination of units A and B can occur in 5% of all events (unconditional probability of 0.05). However, A is common, occurring in 50% of the events, while B occurs in 5% of events. Thus, when B occurs, A occurs in 100% of events (conditional probability: 1), while B only occurs in 10% of events that contain A (conditional probability: 0.1). In this case, the relationship between the two AUs is highly asymmetrical. In facial signals, AU25 (lips part) is necessarily a part of AU27 (jaw drop); however, the reverse is not true. If units have low conditional probability both ways, they are likely unconnected. If both units show equally high conditional probabilities, the occurrence of one of them predicts the other, indicating that they are usually used in combination. For example, AU1 (inner brow raiser) and AU2 (outer brow raiser) are commonly used together in many facial signals (Kohler et al., 2004).

Just as we are interested in the probability between units, we often want to identify the conditional probability that a unit is associated with one condition rather than another. We will refer to this as the *context specificity* of the unit: the probability that the condition is C1 when unit A is observed. For example, in humans, we know that AU9 (nose wrinkle) is strongly tied to the signal of disgust, but very rare in any other conditions, so it has high context specificity, while AU1 (inner brow raiser) is part of many different facial signals (Kohler et al., 2004). For a communicative unit to be tied to a context, it should have high context specificity (only occur in this condition), but also have high probability to occur in this condition.

### NetFACS package

The NetFACS package for R allows users to explore their FACS-like data and test specific hypotheses based on occurrence and co-occurrence probabilities and resampling methods. The package represents the different probabilities of AUs or combinations in the form of undirected (unconditional probability) and directed (conditional probability) networks. Networks can be weighted—the connection between two nodes represents their observed probabilities. Alternatively, networks can be unweighted, only representing connections between two AUs as ‘present’ if they occur more frequently than expected given a null model (Newman, 2010). Networks can represent the connections between two AUs, or the connection between AUs and conditions in the form of bipartite networks.

To test hypotheses using networks, it is essential to construct meaningful null models against which to compare observed data (Farine, 2017). We want to know how much more or less likely the use of an AU or combinations of AUs is than expected, but we have to first define what we expect. Our expectations depend on the question: If we have one large dataset and want to know which AU combinations form predictable units, our expectations are set by the basic probability of each AU to occur on its own. If we compare two conditions (e.g., angry and happy faces), our expectations for the angry faces are set by what would be expected in the happy faces.

In NetFACS, if all events are part of the same condition, expected probability distributions are calculated using pre-network permutations (Farine, 2017): the existing data are shuffled with certain restrictions, to simulate ‘random’ data that still follow some of the underlying rules of the dataset. First, the package extracts the conditional and unconditional probabilities of all possible combinations of AUs in the observed data. To establish whether single AUs occur more or less frequently than expected, the number of AUs in each event (e.g., photo, video frame) is held constant, but which AU occurred is randomised. Thus, if a facial signal contained five AUs before randomisation, it will also do so after randomisation. To establish whether combinations of AUs occur more or less frequently than expected, NetFACS shuffles which AUs occur in each event while keeping the probability of each AU across all events, as well as the number of AUs observed in each event, constant. This randomisation procedure is repeated 1000 times, or any value set by the user. Probabilities for all AU combinations for each randomisation are stored, creating the likely probability space for each combination in the population. The resulting null probability distributions for AU combinations represent the expected data given that AUs were not combined into predictable facial signals. The observed probabilities for the test dataset are compared with the distribution of expected probabilities by calculating a *p*-value (proportion of expected probabilities that are more extreme than the observed value) and effect size (the difference between the observed and average expected value).

If we want to compare two conditions (e.g., old and young participants, or happy and neutral participants), NetFACS uses bootstraps (Carsey & Harden, 2014; Julle-Danière et al., 2020). We use one of the conditions to create the expected distribution for all AUs and their combinations using a bootstrapping procedure (Carsey & Harden, 2014): events from the ‘null’ condition are repeatedly selected randomly with replacement (Efron & Tibshirani, 1993). Probabilities for all combinations are stored, and the procedure is repeated 1000 times (more iterations allow for more stringent tests). As above, the resulting distribution of expected probabilities under the null condition is compared against the observed value in the test condition, and a *p*-value is calculated representing the proportion of expected values that was more extreme than the observed value. By using bootstraps to create null distributions of probabilities for each AU and combination, we do not presuppose normally distributed data (Carsey & Harden, 2014). This bootstrap approach does not make any statements about some unknown population that the data might have been drawn from: it directly compares two empirical distributions. Note that the interpretability of results depends on how well the test and null datasets represent the conditions from which they were drawn: if the probabilities are based on small datasets, the interpretation has to consider this. The more variation there is in the null dataset or the fewer data points we have available, the broader the probability space will be—the expected value becomes more uncertain. Thus, especially for rare elements and noisy data, the resampling approach will potentially favour false negative over false positive values in small datasets.

If individuals provide multiple data points, or videos are analysed, NetFACS allows users to perform the random selection of the bootstrap on the level of the video or individual, so that the null distribution accounts for inter-individual differences and autocorrelation within videos (Harden, 2011). For example, it is possible that only some subjects use specific muscles, in which case the probabilities under the null condition will not be normally distributed. Users can specify control variables (e.g., gender, place of origin), and the selection of events or individuals for the bootstraps will consider these control variables. If the test condition and null condition differ in the composition of the participant pool (e.g., male participants provided more fearful videos), the selection of events for the null distribution maintains the ratio of male and female participants of the test condition, to ascertain that observed differences in the use of AUs are not biased by the sampling effort.

### Networks

For this study, we use networks to answer representative questions on the individual, dyadic, signal, and system level. We will use bipartite, undirected, and directed networks; links between nodes will be either weighted or unweighted (Newman, 2010; see Glossary). Bipartite networks connect nodes of two different categories: in our case, condition and AU. In undirected graphs, the links A ➔ B and B ➔ A are the same. In directed graph, A ➔ B can be different from B ➔ A. In unweighted networks, edges are either present or absent, while edges in weighted networks have different values (in our case, probabilities of co-occurrence). In addition to quantifying dyadic connections, networks allow us to calculate a number of standardised metrics related to the position of the AU in the network—for example its centrality or membership in tight clusters of recurring combinations—and the connectivity of the network as a whole (Croft et al., 2008). In the following, we will describe the networks used for each of the questions; all of these were conducted using the bespoke ‘NetFACS’ package in R, with networks created, analysed, and plotted using the ‘igraph’ package (Csardi & Nepusz, 2006).

## Results

### (a) How specific are AUs to certain stereotypical signals?

#### Method

To understand the strength of the link between AUs and the different conditions, we use a weighted and directional bipartite network of the conditional probabilities of AUs happening given each condition. This tells us how rare or common an AU is in each condition: for example, if the condition is ‘happy’, how likely is it that AU 12 is observed? We also analyse the conditional probabilities of each condition given an AU is observed, indicating how specific the AU is to a condition: for example, if I observe AU12, how likely is it that the condition is ‘happy’? For an AU to be a good signal for a condition, it would have to have a high probability to occur in one condition, but not in others. If an AU is ‘shared’ between different conditions, it is either not indicative as a single AU (but could be context specific as part of a combination), or could be indicative of an underlying dimension that connects conditions (e.g., their valence). If AUs are specific to and common in one condition, they are likely part of a standardised facial signal of this condition. If they are context specific and rare in that condition, their occurrence might indicate a change in meaning, for example if they signal sender uncertainty. The AU might also be very rare and their occurrence in this specific condition might be accidental, or it might have been miscoded. Alternatively, there could be inter-individual differences, and only a subset of individuals displays the AU.

To create the network, we compared the dataset of each condition against all other data points using bootstraps. Thus, to determine whether ‘angry’ faces show specific features at the AU level, we compared the probabilities of AUs occurring in ‘angry’ faces to the null model that AU probabilities across all conditions were the same. Each event was considered independent, i.e., not taken from the same individual or part of a sequence. No information on other variables, such as gender or place of origin, was available in the database we used. Edges between an AU and a condition were assigned if the conditional probability of the AU occurring in this condition was significant at *p* < 0.01, i.e., the observed probability was higher than in 990 out of 1000 bootstraps of the null condition. We represent two distinct graphs for easier comprehension, one quantifying the conditional probability of the AU given the condition (‘Occurrence Probability’) and another quantifying the conditional probability of the condition to the AU (‘Context Specificity’). To test whether distinct ‘clusters’ of nodes existed in the network (signifying groups of AUs and conditions that have strong connections with each other but weak connections to the outside (Girvan & Newman, 2002)), we used the ‘cluster_fast_greedy’ community detection algorithm (Clauset et al., 2004) from the ‘igraph’ package (Csardi & Nepusz, 2006). Modularity values above 0.3 are considered to indicate clear-cut communities of nodes (Clauset et al., 2004).

#### Results

**Fig. 1:**
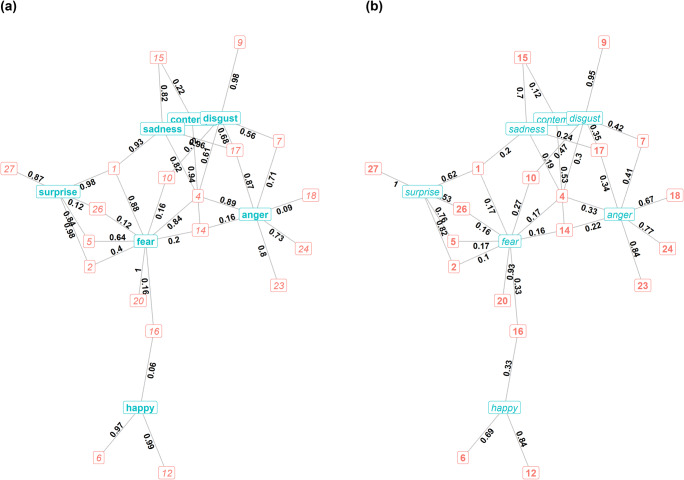
**a** Network representing the probability for any AU to be present in each condition. Only edges that were significantly more likely than expected are displayed (in dark with associated probabilities aside). Squared red numbers are the AUs and conditions are labelled in blue. **b** Network representing the probability for any condition to be shown when an AU was active. Only edges that were significantly more likely than expected are displayed (in dark with associated probabilities aside). Squared red numbers are the AUs and conditions are labelled in blue

The network characterising the occurrence probability of AUs in conditions can be found in Fig. 1a. Only AUs that were significantly more likely than expected to occur in a condition at *p* < 0.01 have links to that condition. The weights of edges between AUs and conditions are the conditional probability of the AU being active in each condition (e.g., the probability that AU4 is active in an ‘angry’ face is 0.89). Figure 1b portrays the concurrent context specificity of AUs (e.g., the probability that the condition is ‘angry’ when AU4 is observed is 0.33).

Combined, the graphs show us that all seven conditions shared some AUs (low specificity, multiple connections), while some AUs were highly informative (high specificity, one connection). For example, as expected, AU6 and AU12 were highly specific to ‘happy’ faces, AU9 to ‘disgust’ faces, and so on, while AU4 was shared by fear, disgust, anger, and sadness. Even though AU4 was not specific to any of these conditions, it occurred at high levels in all of them. Other AUs were specific to a condition but occurred rarely in that condition (e.g., AU26 occurred in 12% of surprised faces, but those were half of all occurrences of this AU). All seven conditions had at least one AU that was both specific to them and highly common (values above 0.7): ‘anger’ – AU23 and AU24; ‘disgust’ – AU9; ‘fear’ – AU20; ‘happy’ – AU12; ‘surprise’ – AU2, AU5, and AU27; ‘sadness’ – AU15; and ‘contempt’ – AU14. The specificity in the latter was slightly lower (0.53) because ‘contempt’ was represented by fewer data points than other conditions. However, all of these connections were probabilistic, not deterministic, as none of them had conditional probabilities of 1 in both directions. This is surprising given that the videos were assigned to each condition based on these AUs.

**Fig. 2:**
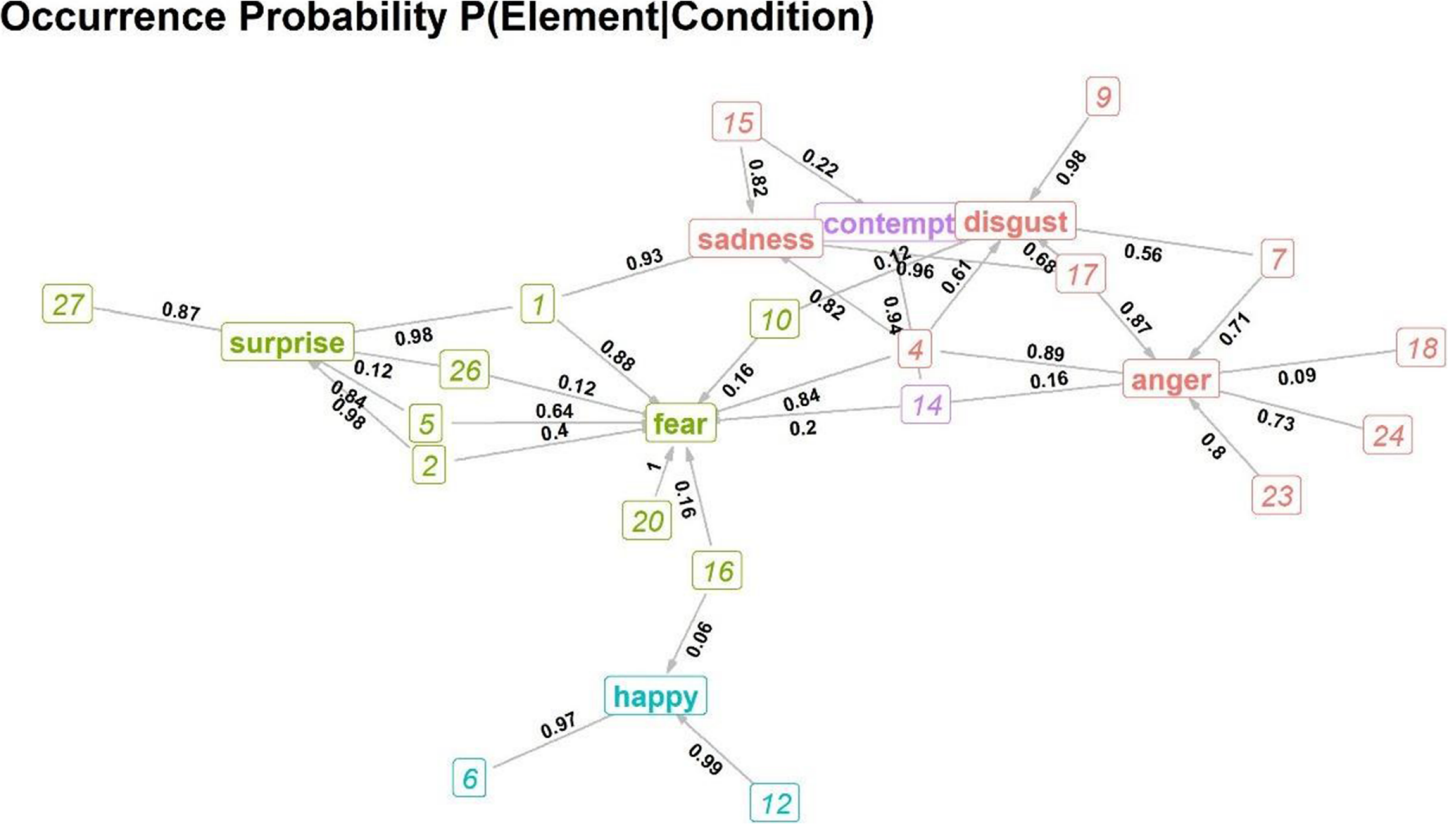
Network representing the probability for any condition to be present when an AU was active. Only edges that were significantly more likely than expected are displayed. Colours represent the different clusters (modularity = 0.50)

Using the community detection algorithm, four clusters were revealed in the bipartite network with a moderately high modularity value (0.50; Fig. 2). ‘Happy’ faces (with AU6 and AU12) and ‘contempt’ (with AU14) formed their own respective clusters. ‘Fear’ and ‘surprise’, which shared a large number of AUs, formed a cluster with AU1, 2, 5, 10, 16, 20, 26, and 27. Lastly, ‘disgust’, ‘anger’, and ‘sadness’, which were linked by the shared use of AU4 and AU17, formed a cluster with those two AUs and AU7, 9, 15, 23, and 24.

### (b) Which AUs are conditional on others?

#### Method

To understand the connections between AUs, we tested their conditional probabilities to occur together. Conditional probabilities were defined by the probability that one AU occurs, given that another one is also present. We explored three possibilities: two AUs can show low conditional probabilities in both directions, in which case they were likely unconnected in the dataset. They can show high conditional probabilities both ways, in which case they formed a functional unit in this condition, with each AU always occurring when the other was present. This connection could indicate that, at least in this condition, they should be treated as one unit rather than two separate units. The third alternative was that conditional probabilities were asymmetrical: if one AU was more common than the other, it is possible that the rare AU always appeared when the more common AU was present, but the same was not true vice versa. These dyads would allow us to identify units that potentially modify meaning, and the cases with and without combinations of the two AUs could be analysed in more detail. For example, in facial signal research, it has long been assumed that adding AU6 (cheek raiser) to AU12 (lip corner puller) modifies the meaning of a smile (Martin et al., 2017; Rychlowska et al., 2017). In that case, when analysing a large number of smiles, we would see a high conditional probability of AU12 occurring when AU6 is present, but not vice versa. Here, we presented the directed weighted network of conditional probabilities of AUs for two conditions in the dataset, ‘sadness’ and ‘surprise’, to exemplify the information that can be obtained by this approach. The weights of each edge represent the conditional probability going each way.

#### Results

**Fig. 3:**
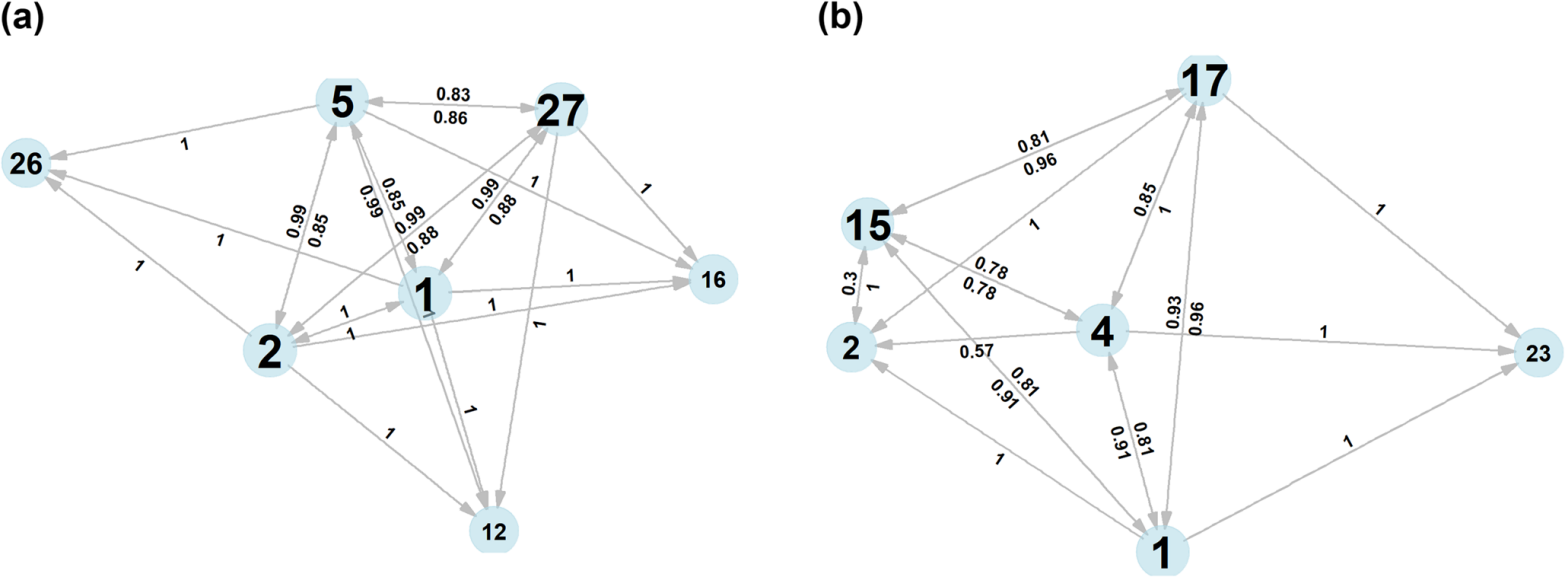
**a** Network representing conditional probabilities of co-occurrence in ‘surprise’. Only connections with probabilities above 0.3 and at least three instances are represented to facilitate comprehension. If conditional probabilities going both ways surpass this threshold, they are represented with two values: above and below the arrow. More common AUs appear larger in the graph. **b** Network representing conditional probabilities of co-occurrence in ‘sadness’. Only connections with probabilities above 0.3 and at least three instances are represented to facilitate comprehension. If conditional probabilities going both ways surpass this threshold, they are represented with two values, above and below the arrow. More common AUs appear larger in the graph

In Fig. 3a and b, we presented the conditional probabilities of AU co-occurrence in two conditions, ‘sadness’ and ‘surprise’. Only connections with a conditional probability over 0.3 and at least three common occurrences are represented in the figures, to facilitate comprehension; this has no influence on the results. AUs with more in- and out-going connections were portrayed larger. The network for ‘surprise’ (Fig. 3a) showed a strong cluster of AU1, 2, 5, and 27 that had high bidirectional conditional probabilities; for each of them, their presence predicted the presence of the others, indicating that they formed a tight unit. AU12, 16, and 26, which also occurred in this condition, were unilaterally tied to AU1, 2, and 5; so in all instances of AU26, these units were also present, but not vice versa. The three rare AUs (AU12, 16, and 26) were not tied to each other, indicating that they occurred in different instances. AU26 and AU27 were not connected, as they cannot be present at the same time in FACS. While ‘surprise’ therefore seemed to have one standardised signal, ‘sadness’ (Fig. 3b) was more variable: AU1 and AU2, which were tightly linked in surprise, were used asymmetrically here: AU1 was more common and not dependent on AU2, which was used in a subset of signals containing the former. AU1, 4, 15, and 17 were used together regularly, but the connections were less deterministic than they appeared for ‘surprise’, with AU4 and AU15 not being tied as strongly as any of the other connections. AU23 replaced AU15 in some circumstances, as it was dependent on the occurrence of AU1, 4, and 17 but never co-occurred with AU15.

### (c) Can we reconstruct the stereotypical facial signals through networks, and how do graphs in different conditions compare?

#### Method

While understanding facial communication on the AU and combination level is valuable, we are often interested in knowing how these units interact to form facial signals. These were clearly defined for some of the data we included in this study (for example, ‘happy’ faces by the presence of AU6 and 12). This will not be the case for all facial signals in all conditions, and displaying the full network of AU combinations can give us a representation of facial activity that combines different information. When representing the outcomes of these analyses graphically, we can represent how often an AU occurred by changing the size of the node in the graph, and we can quantify the strength of the connection between nodes by making stronger edges wider. Strongly stereotyped facial signals will consist of tight clusters of strongly interconnected AUs associated with high computed transitivity (if A → B and A → C, then B → C), while more variable signals should show fewer closed triads and weak connections between larger numbers of AU.

Here, we represented the signal for each condition as an unweighted, undirected network, based on the comparison with all other data points using bootstraps. Thus, a link between two nodes indicates that the two AUs were significantly more likely to co-occur in this condition than across the other conditions. Besides displaying the graphs, we report the network-level centrality measures of density and transitivity (see Glossary for definitions). Density is the ratio of actually existing edges in a network of all possible edges (Newman, 2010); networks with high density have connections between most nodes, indicating either that each facial signal in this condition contains more AUs than in other conditions, or that the AUs are connected more variably. Transitivity refers to the fact that in networks, we often observe that nodes that both have a strong connection to a third node share a strong connection to each other as well (Baronchelli et al., 2013). The network-level transitivity measure or clustering coefficient quantifies the probability that two nodes were connected if they had a node in common. Highly transitive networks are clustered, which would be the case if all AUs that occurred were connected to all others (if density is high), or if the network consisted of two sets of AUs that can express the same information content (low density). Networks with low transitivity show many AUs that are isolated or just connected to one other AU.

#### Results

**Fig. 4:**
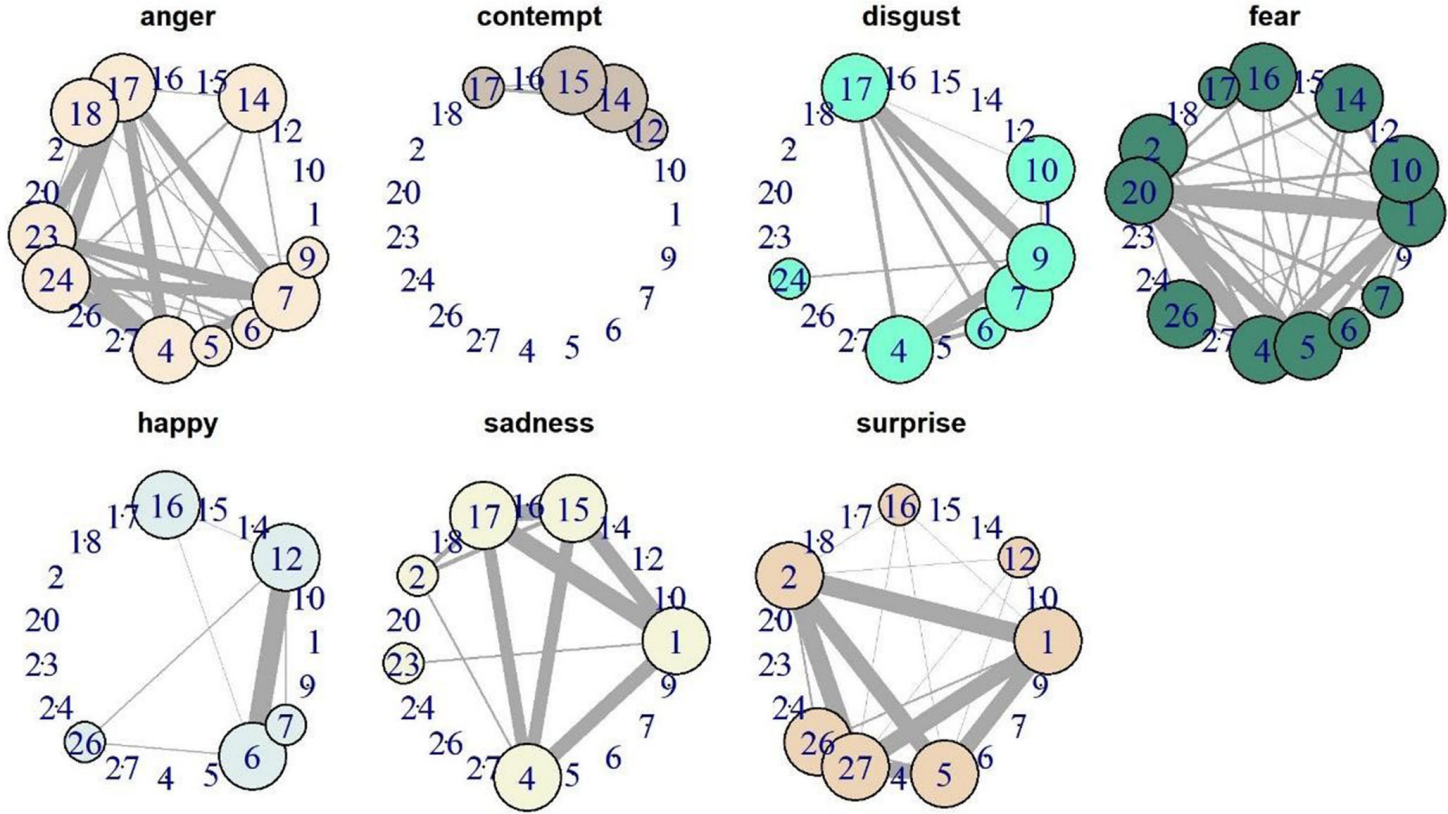
Network graphs for all seven conditions. Only significant connections are portrayed; thicker edges indicate higher probability of co-occurrence. Large points indicate AUs that are significantly more common than expected in this condition, small points indicate AUs that were observed but not significant, no point indicates that the AU was not observed in this condition

Figure 4 represents the networks for each condition, with significant connections between AUs used as edges and AUs that occurred significantly more often than expected as larger points. Connections between AUs were significant if they occurred at a higher probability in this condition than would be expected across the other conditions. Small points were nodes that occurred in the condition, but not significantly more than expected. The graphs showed that in this dataset, each of the conditions consisted of a core of AUs that were common and strongly linked to each other, corresponding to the known facial signals (Ekman & Oster, 1979): ‘happy’, for example, consisted of AU6+12, ‘sadness’ of AU1 + 4 + 15 + 17, and so on. However, this way of plotting the data also revealed that even in this posed dataset, there was variation within facial signals, and a number of rare AU accompanied at least a subset of signals in each category. This was especially clear in ‘fear’, which consisted of a core of AU1 + 2 + 4 + 5 + 20, but was often accompanied by AU10, 14, 16, 17, and 26. That all of these different combinations were still recognised as the same condition when the dataset was compiled indicates that facial signals are relatively robust to variation in AU activity.

Besides plotting connections using graphs, we can represent information about the network structure using different centrality measures. Here, we exemplified this by using the density of the graph (number of edges compared to number of possible edges) and the transitivity (ratio of closed triads; Table 1). ‘Happy’ faces (0.04) and ‘contemptuous’ faces (0.03) were the least dense, indicating that they employed only a very small number of possible combinations of AUs, which is also visible in Fig. 4. These facial signals were therefore simpler and more stereotypical than the others. ‘Fear’ (0.23) and ‘anger’ (0.15) were the densest graphs, thus using the highest number of different units, making them potentially the most complex signals in this dataset. While ‘surprised’ faces were relatively dense, they also had a high number of closed triads (transitivity = 0.81), indicating that the AUs that did get used, were used all together most of the time. This stood in contrast to ‘happy’ and ‘contemptuous’ faces, where only two AUs were strongly connected respectively, making strong triads unlikely. Again, ‘fear’ stood out by its low transitivity value, indicating that while many AUs can be active in this condition, they did not occur together, creating open triads.

**Table 1:** Network summary statistics for the seven conditions

	Number of nodes	Number of edges	Density	Transitivity
Anger	18	23	0.15	0.78
Contempt	18	4	0.03	0.60
Disgust	18	14	0.09	0.78
Fear	18	35	0.23	0.56
Happy	18	6	0.04	0.55
Sadness	18	10	0.07	0.78
Surprise	18	17	0.11	0.81

### (d) Can we identify clusters of AUs without knowledge of the underlying conditions?

#### Method

While here we are working with a dataset where clear conditions are present in the form of seven stereotypical signals, this will not always be the case when using FACS: especially when working with non-human animals or large computer-coded datasets of spontaneous signals, clear-cut categories might not be forthcoming. In those cases, it would be useful if the connections between AUs could identify the underlying structure of the communication system.

Here, we pretended that we did not know the underlying structure of the dataset, and used a community detection algorithm on the full dataset to detect clusters of AUs that co-occurred more than expected, to see whether we could identify the conditions. We used a weighted and undirected network (i.e., connections between AUs are represented by how often they co-occur). We used the ‘fast greedy’ modularity optimisation algorithm for finding community structure implemented in igraph (Clauset et al., 2004), which divides the network into communities based on modularity by assigning nodes to clusters that minimise the edges between clusters and maximise edges within clusters (Newman & Girvan, 2004). Random distribution of edges would be associated with modularity values of 0; complete separation between clusters would show modularity of 1. A modularity value above 0.3 is a good indicator of meaningful community structure (Clauset et al., 2004). Above, we saw that a similar approach for the bipartite network including the conditions and AUs they were significantly associated with, detected four clusters.

#### Results

**Fig. 5:**
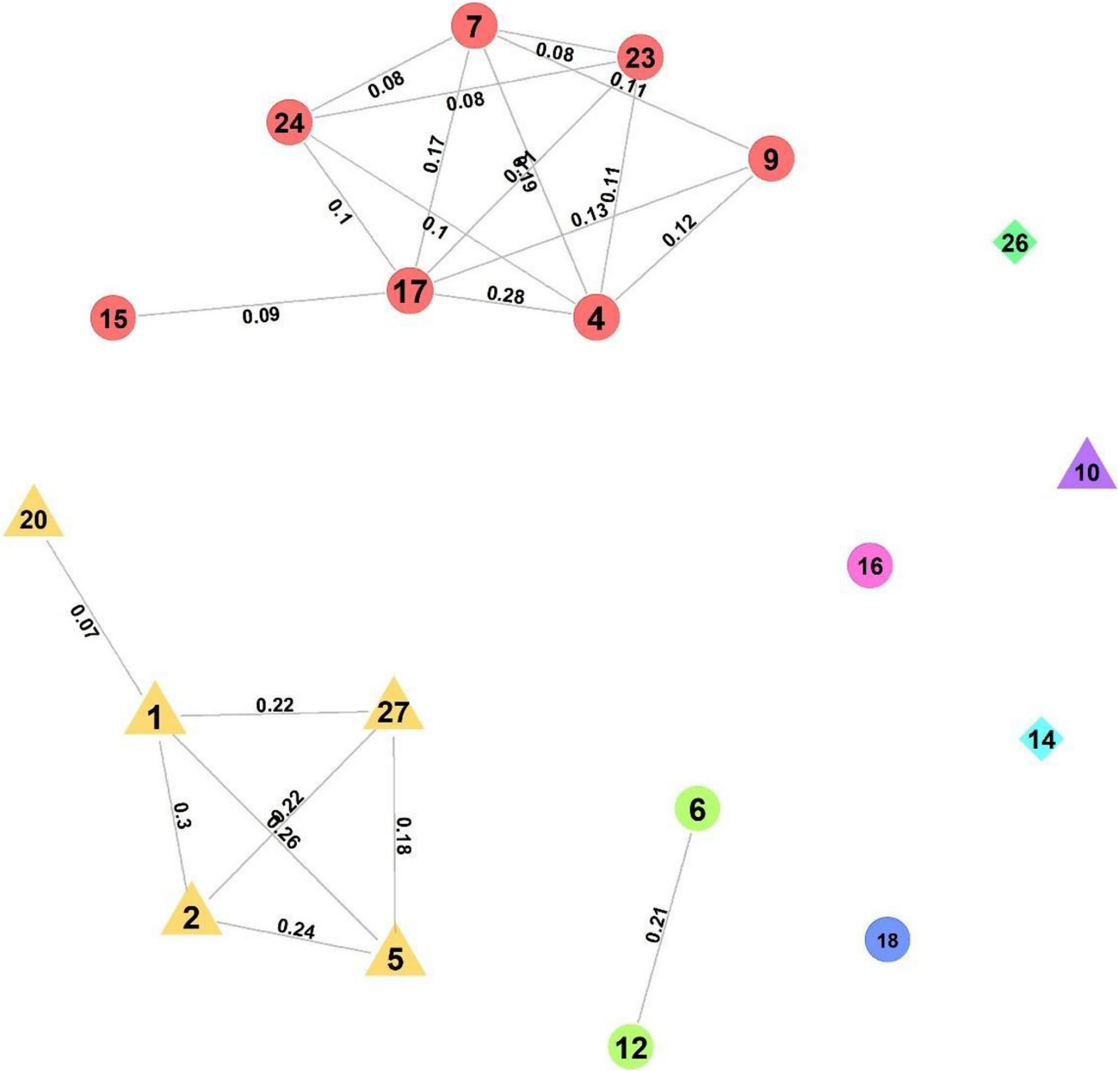
Graph representing data combining all conditions, with colours representing the different clusters identified by the algorithm. Clusters have higher connections within than without. Modularity was 0.49, indicating clear clusters

Based solely on the links between AUs without contextual knowledge, the algorithm detected three clusters with a modularity of 0.49 (Fig. 5). The first cluster, including AU6, and 12, is equivalent to the ‘happy’ cluster above. A second cluster, including a core of AU1, 2, 5, and 27, was largely equivalent to the surprise cluster detected before. AU 20 was connected to AU1, because of their strong connection in the ‘fear’ condition. The largest cluster, containing AU4, 7, 9, 15, 17, 23, and 24, represents the previous cluster for angry/disgusted/sad faces. The best indicator for contempt (AU14) was not part of a cluster, because it did not have strong connections with any other AU in that condition and ‘contempt’ itself was much rarer than the other conditions. AU10, 16, 18, and 26 were not consistently connected to any of the other clusters. Thus, even without knowledge about the conditions underlying the facial signals, we would be able to detect that there is a highly stereotypical signal containing AU6 + 12. We would be able to distinguish between signals with lowered (AU4) and raised (AU1 + 2) eyebrows, which was a central distinction in this dataset. However, the results also demonstrate how the accuracy of this method relies on sufficient available data for all conditions.

## Discussion

In this article, we introduce network science as a tool for the analysis of facial signal data. The Facial Action Coding System (FACS) provides a way to study facial communication in immense detail, but the data it produces have many properties that make analysis difficult. As a result, the data generated from FACS measurement are often highly underused. Networks have been used widely in communication research in humans and animals (Allen et al., 2019; Kershenbaum et al., 2016; Lynn et al., 2020; Peng et al., 2008; Weiss et al., 2014), due to their flexibility in answering various questions on the unit level, combination level, and the level of the whole communication system (Lynn et al., 2020; Newman, 2010). Here, we test their potential use for the study of facial signals, showing that they allow us to gain new insights even when applied to well-studied datasets (Lucey et al., 2010).

All results presented here regarding the use of AUs and combinations in different stereotypical facial signals emphasise different aspects of the face as a communication system. All seven signals contained some AUs that were highly context specific and mainly occurred in this condition. However, these AUs were not used in isolation, with most conditions sharing AUs with others. These shared AUs were largely centred on the brows: conditions clustered into those with raised eyebrows (‘surprise’, ‘fear’) and those with lowered eyebrows (‘anger’, ‘sadness’, ‘disgust’); those without strong eyebrow activity (‘happy’, ‘contempt’) formed their own clusters. This lack of specificity of eyebrow movements might explain why some observers seem to prefer using the mouth region to distinguish between conditions (Blais et al., 2012), even though this is not a culturally universal feature (Jack et al., 2012). These differences can be replicated partially without knowledge of the original conditions, evidence for the ability of the network approach to recreate the structural features underlying large datasets with unknown properties. Looking beyond the use of single AUs, the network approach revealed that important information is contained in the combination of AUs and their conditional probabilities. For example, while both ‘sadness’ and ‘surprise’ show the use of AU1 and AU2, these were strongly linked in surprised faces, while AU2 is only sporadically used in sad faces.

The Extended Cohn-Kanade Database (Kanade et al., 2000; Lucey et al., 2010) uses highly stereotypical signals, which is not representative of the expressivity and flexibility of human facial behaviour. The network approach visualises that beyond the standardised signals, other AUs are often active in a subset of signals, while the ‘stereotypical’ AUs were only probabilistically connected to each condition. Our perception of facial signals therefore seems to be robust against changes in the setup of units that we observe. The facial signals in conditions differed in their overall complexity: ‘happy’ and ‘contemptuous’ faces were highly stereotypical, with very low network density and very few AUs used. This is partially due to their consistent lack of eyebrow movements, which limits the number of AUs that can be involved. In the ‘fear’ signals, on the other hand, many AUs were added to the core cluster of units at different times. Information-centred approaches to quantifying complexity (such as the information entropy in each condition) could be used to understand why signals differ in flexibility and what effect this has on receivers. Larger, more diverse and naturalistic datasets would be needed to test whether the inclusion or exclusion of AUs and combinations represent inter-individual differences or affects the meaning of the signal (Feldman-Barrett et al., 2019; Julle-Danière et al., 2020; Waller et al., 2020).

Networks can be tailored to answer different questions, but most studies will be interested in the same general patterns: which AUs are used more in some contexts than others, and how informative is their use? How do these AUs combine to form meaningful signals? Do all AUs in a context matter, or is there a core of highly specific units? Does the addition of some AUs change the meaning of a facial signal? How do inter-individual differences or other factors, such as culture, influence the use of facial signals? The NetFACS package allows users to answer these questions, and many others, in a standardised and statistically meaningful way. While we defined network edges in this study using the co-occurrence of AUs in the same signal, one strength of the network approach is its flexibility when applied to different questions. Importantly, using transitions between AUs and AU combinations within sequential FACS data would provide an important step towards understanding the face as a communication system (Kershenbaum et al., 2016). Our results further confirm that single AUs are probably poor indicators of a single meaning, and not every AU in a signal necessarily changes its meaning. Thus, preferably, there would be a two-pronged approach for network analysis of facial signals: use networks to identify specific units and then use transition networks to test how these units connect to each other (Allen et al., 2019; Sasahara et al., 2012; Weiss et al., 2014). Dynamic networks and transition networks—between AUs and full signals—will be a central feature of future development of the NetFACS package. One question is sample size: the permutation and bootstrap approaches should be relatively robust, but datasets need to be representative of the complexity of facial signals in the context in question. The more varied signals in a context are, the more data will be necessary to accurately depict their probability space—especially for AU combinations beyond the dyadic (the focus here), and for dynamic signals. The other question is the validity of the AU coding—human coding is tedious and slow and often prevents the analysis of large-scale datasets, while automatic decoders still lack strong performance. However, the network approach potentially allows for the analysis of noisy data, as uncertainty of expected probability distributions is encoded and interpretable.

The code underlying the package is openly available, allowing users to develop their own algorithms for inclusion in future versions of the package. Importantly, the package is also of use to researchers studying other aspects of communication; the only requirement currently is that data can be coded as matrices of presence/absence of units at certain events. Units can be defined by the researcher as letters of the alphabet, syllables, words, or animal gestures or calls; as long as questions can be framed around the occurrence and co-occurrence of units, NetFACS can be used to conduct statistical analyses into the structure of the communication system.

Facial signals are a vital aspect of the communication systems of many mammal species (Waller & Micheletta, 2013); the development of Facial Action Coding Systems for different species has created the potential to directly compare the use of facial signals and the complexity and flexibility of facial communication with an evolutionary framework (Waller et al., 2020). NetFACS offers a way to analyse the wealth of data within and between species, as well as within and between individuals. Even if the AUs themselves differ, the system approach underlying networks puts a focus on connectivity and flexibility of unit combinations. It therefore creates a vocabulary to describe the information content inherent in the face as a communicative tool. As the complexity of networks can be formally described, we compare the complexity of communication systems of different species and study the evolution of facial communication and the antecedents for the rise of more complex facial communication in some species (Rebout et al., 2020). Other communication systems, if framed similarly as networks of co-occurring or sequential elements, could be analysed in comparison with facial signals, or different systems could be combined to better understand multimodal communication (Slocombe et al., 2011). Thus, as a statistical package, NetFACS offers analytical tools for a broad range of researchers studying communication from an evolutionary standpoint.

## Data Availability

Data and scripts are available as part of the NetFACS package. The GitHub repository contains a tutorial and all data used for this study: https://github.com/NetFACS/NetFACS
